# Urban Form and Function Optimization for Reducing Carbon Emissions Based on Crowd-Sourced Spatio-Temporal Data

**DOI:** 10.3390/ijerph191710805

**Published:** 2022-08-30

**Authors:** Fangjie Cao, Yun Qiu, Qianxin Wang, Yan Zou

**Affiliations:** 1 School of Environment and Spatial Informatics, China University of Mining and Technology, Xuzhou 221116, China; 2 School of Humanity and Law, Beijing University of Civil Engineering and Architecture, Beijing 102616, China

**Keywords:** low-carbon urban planning, Landscape Shape Index, POI, OSM, CO_2_ concentration

## Abstract

The low-carbon city has become an important global urban development-oriented goal. One important aspect of urban space is low-carbon urban planning, which has a vital role in urban carbon emissions. Which types of urban form and function allocations are conducive to reducing carbon emissions is therefore a key issue. In this study, the Futian and Luohu Districts of Shenzhen, Guangdong Province, China, are taken as an example to investigate this issue. Firstly, a “head/tail” breaks method based on the third fractal theory is adopted to obtain the minimum evaluation parcel of urban space. Then, the Landscape Shape Index (LSI), Fragmentation Index (C), Shannon’s Diversity Index (SHDI), and Density of Public Facilities (Den) are used to evaluate the form and function allocation of each parcel. In addition, the CO_2_ concentration distribution in this study area is acquired from remote sensing satellite data. Finally, the relationships between urban form, function allocation, and CO_2_ concentration are obtained. The results show that the lower the urban form index or the higher the urban function index, the less the CO_2_ concentration. To verify this conclusion, three experiments are designed and carried out. In experiment A, the CO_2_ concentration of the tested area is reduced by 14.31% by decreasing the LSI and C by 6.1% and 9.4%, respectively. In experiment B, the CO_2_ concentration is reduced by 15.15% by increasing the SHDI and Den by 16.3% and 12.1%, respectively. In experiment C, the CO_2_ concentration is reduced by 27.72% when the urban form and function are adjusted in the same was as in experiments A and B.

## 1. Introduction

Global warming has become a serious challenge to mankind, leading to sea level rise, the loss of coral reefs, and a variety of natural disasters. CO_2_ makes up approximately 70% of the greenhouse gases, which are considered to be one of the greatest causes of global warming [1]. However, due to rapid global urbanization, more than 76% of CO_2_ emissions come from cities [2]. Therefore, low-carbon urban planning and development has attracted more and more attention in recent years [3,4]. In general, current research on urban carbon emissions focuses on two aspects. One is the calculation of models of urban carbon emissions, such as the multi-regional input–output (MRIO) model, the single-regional input–output model (SRIO) [5], the multi-scale input–output model (MSIO), and the IPCC carbon emission calculation method [6]. The other is analysis of the leading factors of urban carbon emissions, such as index decomposition analysis [7], Kaya identity [8], and the environmental input–output analysis framework [9]. These studies show that population size, investment intensity, and land use change have significant effects on urban emissions [10,11,12].

In addition, urban spatial structure, function allocation, and social organization also have an important impact on carbon emissions. Therefore, they have become important issues in low-carbon urban planning. For example, Fang [13] investigated the relationship between landscape index and CO_2_ emissions by the remote sensing image data of 30 provinces in China from 1999 to 2019. The results showed that urban expansion leads to an increase in CO_2_ emissions, and that the CO_2_ emissions of irregular urban expansion are more than those of aggregated and continuous urban expansion. For another example, Ou [14] analyzed the impact of different urban development models on carbon emissions. It was verified that the multi-core urban model has less carbon emissions than the single core urban model. In addition, Yang [15] discussed the relationship between the urban morphology and the carbon emissions and pointed out that urban traffic carbon emissions can be reduced by cutting down the population density, the built-up area, and the road density.

These studies are helpful for revealing the leading factors of urban carbon emissions and optimizing the urban development model. However, they only focus on the influencing factors and mechanisms of urban carbon emissions at a large scale (e.g., province and city), and there is a lack of research at smaller scales (e.g., district, street). One of the key limiting factors is the difficulty of data acquisition for small-scale carbon emission research, especially the fine statistical data of social economy and carbon emissions. However, the increase in crowd-sourced spatio-temporal big data provides a good opportunity to alleviate this problem [16]. They are easier to obtain, faster to update, finer in scale, and lower in cost than traditional statistical data [17,18]. For example, OpenStreetMap (OSM) has been widely used to divide the minimum evaluation parcel in urban quantitative research, due to the accuracy and integrity of road network data [19,20]. For another example, Points of Interest (POI) has often been used to identify urban function zones by marking the attribute information of each parcel [21,22]. In addition, mobile phone signaling data [23], bus card data [24], taxi trajectory data [25], and other crowd-sourced spatio-temporal data [26] have also been used to study the characteristics of urban structure, function, and operation.

In this study, OSM data are used to divide minimum evaluation parcels, and a “head/tail” breaks method is adopted to improve the rationality and practicality of the parcel division results. POI data are used to determine the function of each parcel, and the CO_2_ concentration distribution is also acquired from Landsat-8 remote sensing image data. The spatial form and function allocation of each parcel is evaluated by the Landscape Shape Index (LSI), Fragmentation Index (C), Shannon’s Diversity Index (SHDI), and Density of Public Facilities (Den). Finally, the relationships between urban morphology and CO_2_ concentration are constructed. The results indicate that regular form, low fragmentation, and perfect function all benefit the reduction of urban carbon emissions. Furthermore, several simulation experiments are designed and carried out to compare the effects of different planning schemes on urban emissions. It is verified that the CO_2_ concentration of tested areas can be reduced by decreasing the LSI and C or increasing the SHDI and Den.

## 2. Study Area and Data Sources

### 2.1. Study Area

**Figure 1:**
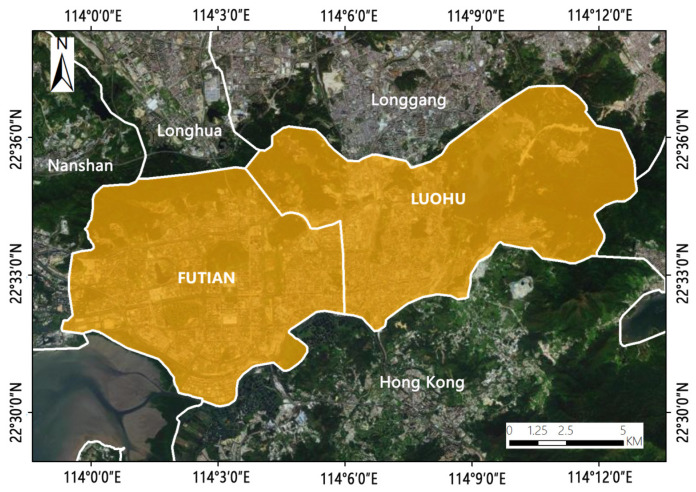
Schematic diagram of the study area.

This study was carried out in the Futian District and Luohu District of Shenzhen City, which is located in the Guangdong Province in southern China, adjacent to Hong Kong, with an area of 11.541 km^2^ and a population of approximately 270 million (Figure 1). It covers 0.2% of the country and was established as the first special economic zone and contributes 2.7% of China’s Gross Domestic Product (GDP). Moreover, Futian District is the administrative, financial, cultural, and commercial center of Shenzhen.

The research area is expected to transform into a sustainable area. In the past few years, energy structural changes, industrial structural improvement, and green city construction occurred in Shenzhen. Today, Shenzhen’s carbon intensity has continued to decline. In particular, the green coverage area in Luohu District is 51.66 km^2^, and the green coverage rate is 64.5%, which makes Luohu District an important carbon sink area in Shenzhen. Thus, Futian District and Luohu District could be an ideal research area to provide reference samples of low-carbon urban planning to analyze the change of CO_2_ concentration under the adjustment of landscape pattern.

### 2.2. Data Sources

In this study, there are three types of data collected and used: one is the POI data from 2022, which are used to calculate landscape index; the second is the road network data from 2022, which are used to delineate landscape parcels; and the third is Landsat-8 image data, which are used to estimate CO_2_ concentration distribution. Considering the season and cloud cover, the Landsat-8 image data from 2021 were collected (a list of data sources is shown in Table 1).

#### 2.2.1. POIs Data from Amap

**Figure 2:**
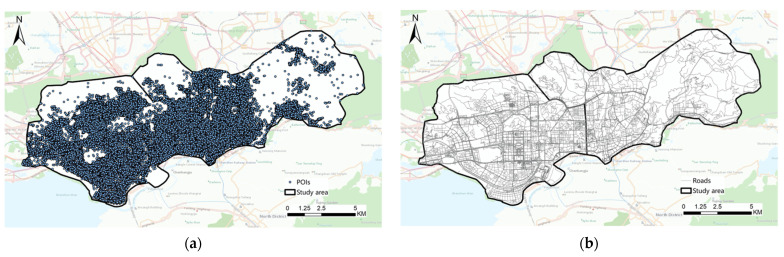
Schematic diagram of data distribution in the study area. (**a**) POIs spatial distribution. (**b**) OSM spatial distribution.

POIs generally refer to all geographical objects that can be abstracted as points and are mainly used to represent various service facilities in urban spaces. POI data cover spatial distribution and attribute the information of geographical objects, which are widely used in urban spatial structure and function analysis [27,28,29], vitality evaluation [30,31], and land cover verification [32,33]. POIs can be easily obtained by a special software development kit (SDK) provided by the Amap open platform (https://lbs.amap.com/, accessed on 1 January 2022), the acquisition methods of which include key-word search, peripheral search, ID search, etc. In this study, the polygon retrieval method is adopted to obtain POI data. Finally, a total of 248,968 POI data are obtained, the spatial distribution of which is shown in Figure 2a.

#### 2.2.2. OpenStreetMap Road Network Data

The urban road network is a vector structure composed of items of a certain density and appropriate form. It is also an important way of separating urban space parcels. The road network data are provided by OpenStreetMap (OSM, https://www.openhistoricalmap.org/, accessed on 1 January 2022), which is an open-source project created free of charge. OSM is one of the most accurate and complete vector geographic datasets and includes user-generated maps of every part of the world. The road network data in this study were obtained from OSM in the Futian District and Luohu District of Shenzhen and include 12 types of road grades. The spatial distribution is shown in Figure 2b.

#### 2.2.3. Landsat-8 Remote Sensing Image Data

The remote sensing image data used in this study are Landsat-8 remote sensing data, which are provided by the National Aeronautics and Space Administration (NASA, https://www.nasa.gov/, accessed on 20 February 2021). Landsat-8 is the eighth satellite of the US Landsat program (Landsat). The Landsat-8 satellite payload consists of two instruments: Operational Land Imager (OLI) and Thermal Infrared Sensor (TIRS). The OLI provides nine bands at a spatial resolution of 30 m. TIRS provides two separate thermal infrared bands at a resolution of 100 m. Landsat series data are currently widely used in many research fields, such as natural resource protection [34], energy exploration [35], environmental management [36], and natural disaster monitoring [37].

The monitoring of urban CO_2_ concentration changes is based on satellite thermal infrared datasets. It has low spatial resolution based on the current data from Advanced Very-High-Resolution Radiometers (AVHRR). In addition, it is impossible to describe the refined surface temperature of a small area. Therefore, this study chooses Landsat-8 data and uses the single-window algorithm to estimate the change of Land Surface Temperature (LST). Through the analysis, the relationship between LST and actual CO_2_ concentration is obtained, and then the distribution characteristics of CO_2_ concentration are revealed.

## 3. Methods

**Figure 3:**
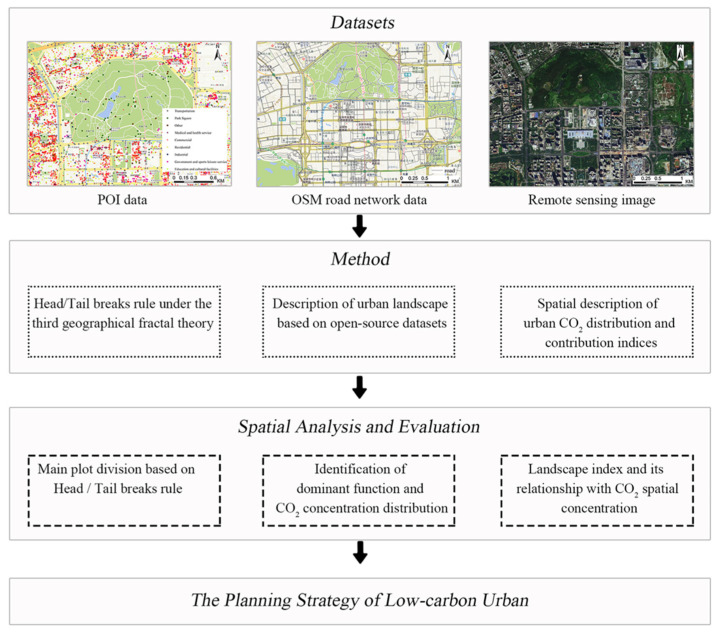
Overall framework.

The proposed framework is displayed in Figure 3. The framework consists of three components. First, the third fractal theory is introduced to divide the urban space and establish a new urban evaluation parcel, which is described in Section 3.1. Then, open-source datasets (including POI and OSM road networks) are used to characterize urban landscapes morphology in Section 3.2. Third, in Section 3.3, urban CO_2_ concentration is extracted using Landsat-8 data, and the relationship between urban form and CO_2_ concentration is analyzed based on a contribution model.

### 3.1. Head/Tail Breaks Rule under the Third Geographical Fractal Theory

There are two important scientific problems in the division of ranking of traditional urban research datasets. One is the number of ranks, and the other is the width of the interval number. Most of the current classification methods are only suitable for data that conform to linear distribution characteristics, such as equal interval, quantile, geometric interval, and standard deviation [38]. However, the urban development pattern is not linear and is characterized by a power-law distribution. For example, the size of cities, the characteristics of urban population clusters, and even the primacy of larger urban agglomerations all follow a unique pattern of heavy “tail” distribution [39,40].

**Figure 4:**
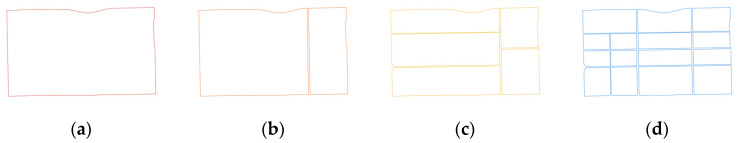
Induction pattern of natural street parcels. (**a**) initial state. (**b**) 1st breaks. (**c**) 2nd breaks. (**d**) final state.

To solve the above problem, we introduced a third fractal approach (head/tail breaks method [41,42]) under the natural city theory, which helps to divide the landscape parcels without destroying the existing structure of the city. This is a bottom-up urban structure detection method, which can automatically detect all the smallest substructures and merge them into a larger parcel. Thus, the head/tail breaks method is adopted to obtain the evaluation parcels in this study (Figure 4). Firstly, we build a natural street network model based on OSM streets, choosing all roads and treating each one as an independent individual. Secondly, the outermost ring-road is regarded as the most important element for dividing the study area. Then, sub-periphery roads are considered as sub-important elements for continued segmentation of the study area, and this is continued until the smallest evaluation parcel is divided. In other words, when there are roads of the same importance level in a certain induction, the division of evaluation parcels is stopped immediately.

### 3.2. Description of Urban Landscape Based on Open-Source Datasets

#### 3.2.1. Identification of Dominant Function with Semantic Association and LSTM Model

There are several problems in using POIs to identify dominant function. First, the word count of POIs that attribute information (a type of Extremely Short Text) is limited; second, the model is easy to disturb, which results in poor classifier performance; third, the POI data types are complex, and the classification standards are not unified; last, the name and type in the POI attribute are inconsistent; for example, the parcel whose name ends in a garden is classified as a park.

To solve these problems, a multi-level short text classification model that integrates semantic association and Long Short-Term Memory (LSTM) [43] is adopted in this study. We use semantic association to extract the name information of POIs, establish its relationship with function classification to identify the urban function (classification criteria is shown in Table 2), and apply LSTM for supervised classification. In addition, the training set and validation set are divided according to the ratio of 4:1; the deep learning framework is pytorch1.7.0 and the GPU is GeForce RTX 3070.

#### 3.2.2. Urban Form Description Using Landscape Index

In this section, urban landscape pattern is used to describe urban morphology. Landscape metrics analysis is a widely used method to describe the structure of landscapes. From the scale of measurement, the landscape indices are generally divided into patch-level indices, class-level indices, and landscape-level indices. To measure the morphological and function characteristics of the entire study area, LSI, C, SHDI, and Den are selected. LSI and C are calculated to describe urban spatial form.

Notably, we cluster POI data of the same type in each landscape parcel and outline its range line to form the region of interest (AOI), then calculate the indices based on AOI. In addition, SHDI and Den can reflect the function diversity and spatial agglomeration characteristics and measure the service capacity of the evaluation parcel. The calculation formula is shown in Table 3.

### 3.3. Spatial Description of Urban CO_2_ Distribution and Contribution Indices

#### 3.3.1. Spatial Distribution of Urban CO_2_ Concentration

The single-window algorithm is used to estimate the LST. In addition, to verify the accuracy of the inversion results, we compare the measured air temperature at 31 test points with the inversion LST. After analysis, it is found that the inversion LST has a very high correlation with the measured temperature, and the correlation coefficient, R, is 0.831, which means that the LST is basically consistent with the actual situation.

For establishing the relationship between CO_2_ concentration and LST, we measure the CO_2_ concentration at 20 test points, the relationship equation is established by regression analysis [44], and the fitting effect reaches 0.896.

#### 3.3.2. Description of Spatial Distribution of Urban CO_2_ Concentration

Carbon emissions are related to urban form, but small-scale carbon emissions cannot be finely spatially measured. Therefore, we consider the CO_2_ concentration instead of carbon emissions. In order to quantitatively analyze the contribution of each landscape index to CO_2_ concentration, the contribution indices of different landscape indices to CO_2_ concentration are calculated [45,46]. The formula is as follows:

(1) $$CI_{ij}=T_{i}\times \frac{I_{ij}}{\bar{I_{j}}}$$

where $CI_{ij}$ is the contribution index of landscape index j to CO_2_ concentration in parcel i; $T_{i}$ is the difference between the average CO_2_ concentration in parcel i and the average CO_2_ concentration in the study area; $I_{ij}$ is the value of landscape index j in parcel i; $\bar{I_{j}}$ is the average value of the landscape index j in the study area; i is the ID of the evaluation parcel.

## 4. Experiments and Analysis

### 4.1. Main Plot Division Based on Head/Tail Breaks Rule

**Figure 5:**
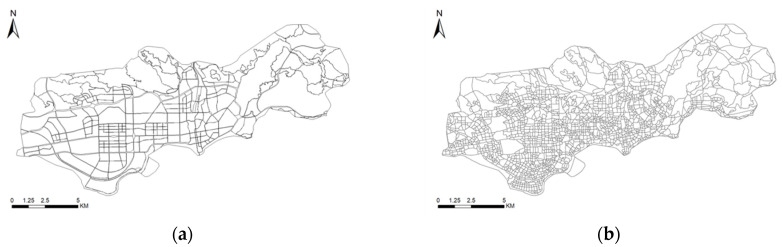
Evaluation parcel under two segmentation methods. (**a**) the scaling form under the head/tail breaks method. (**b**) the minimum evaluation parcel presented with the traditional “thinning expansion” segmentation method.

In this section, a new method for detecting the minimum evaluation parcel for urban planning is applied. The “Head/tail” breaks method is proposed for the segmentation of urban landscapes, and the results are shown in Figure 5a. Figure 5 also presents the comparison between the head/tail breaks method and the traditional segmentation method. Figure 5a represents the scaling form under the head/tail breaks method, where the evaluation parcel can be automatically detected; Figure 5b represents the minimum evaluation parcel presented with the traditional “thinning expansion” segmentation method. It is different from the traditional separation parcel, which only relies on the road network for “thinning expansion”, and the results are more fragmented. The result of the head/tail breaks method integrates more and smaller “substructures”, which makes the research area form a structured whole. In other words, in this research, an evaluation parcel consists of small substructures rather than large ones. The study area is divided into 285 landscape parcels (the traffic and transportation dominated by roads are connected in series into a complete landscape parcel). Under the head/tail breaks method, each functional subject is basically completely divided in a landscape parcel. In contrast to the mechanical segmentation method, the “head/tail” breaks method used in this study allows for the direct use of major roads to divide traffic functional areas.

### 4.2. Identification of Main Functional Areas and CO_2_ Concentration Distribution

**Figure 6:**
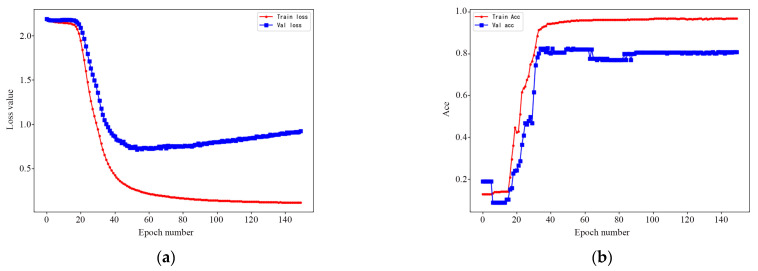
Training and testing efficiencies. (**a**) training efficiencies. (**b**) testing efficiencies.

With the primary aim of distinguishing the functions in each urban landscape parcel, we reclassify the 23 one-level types of Amap POI data into nine types, including commercial, residential, government and sports leisure service, education and cultural facilities, medical and health service, park squares, transportation, industrial, and other. In this paper, the LSTM Extremely Short Text classification network is used to extract elements. The network training learning rate is 0.00003, the number of iterations is 150, and batch size is 64. In particular, the prediction accuracy of the POI classification function model is as high as 85.39%. Figure 6 shows the training and testing efficiencies (In Figure 6a, the x represents the training period and the y represents the accuracy and loss function value of the network in the training stage. In Figure 6b, y verifies the accuracy and loss function value of the network in the network performance stage). The red curve represents the training stage, and the blue curve represents the verification stage. The weight file with the highest accuracy in the training process is used to generalize more than 240,000 dataset files, so as to obtain the classification results of all POI data in the study area (the classification results of POIs are shown in Table 4), thus providing a database for the subsequent analysis.

The Term Frequency–Inverse Document Frequency (TF–IDF) algorithm combines spatial correlation and linear regression methods and is utilized to improve the traditional models [47,48]. Compared with the traditional models, such as determining the type according to the number of POIs, using the TF–IDF algorithm to deduce the leading function of a landscape parcel through frequency density has certain advantages, because it can judge the dominant function of the landscape parcel with mixed functions by calculating the weight of POIs. The model structure is as follows:

(2) $$TFIDF=\frac{n_{i,j}}{A_{i}\sum_{k}n_{k,j}}\times \log\frac{1}{\left|j:t_{i}\in A_{i}\right|}$$

where TFIDF is the weighted value of POI type i in landscape parcel j; i is POI type; j is the landscape parcel; $n_{i,j}$ is the number of occurrences of POI type i in landscape parcel j; $\sum_{k}n_{k,j}$ is the total number of POI occurrences for landscape parcel j; and $\left|j:t_{i}\in A_{i}\right|$ is the total number of landscape parcels containing a certain type of POI.

**Figure 7:**
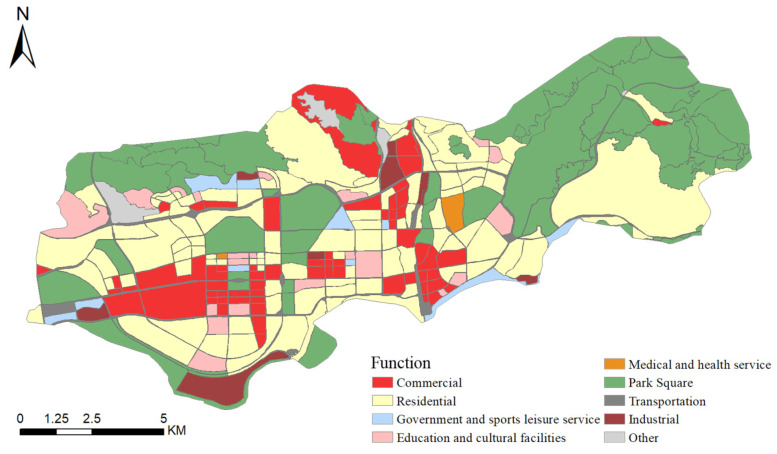
Dominant function partition of study area.

The spatial distribution of the results of the dominant functional division of the study area is shown in Figure 7. From the perspective of the proportion of functional parcels, residential, commercial, and park squares rank in the top three of the dominant functions, accounting for 34.84%, 23.34%, and 22.56%, respectively. From the perspective of area ratio, the ranking is residential—park square—commercial, and park square and the first dominant function (residential) parcel area ratio is very close, respectively, 33.81% and 34.33%. The commercial functional area accounts for 10.81% of the area, only 0.39% more than the fourth-ranked traffic functional area. In general, the study area has a complete urban function, a high proportion of open spaces, and a well-connected transportation network. In other words, the study area is efficiently and intensively utilized with minimum area and time cost, which provides the conditions to ensure the green development of the city.

**Figure 8:**
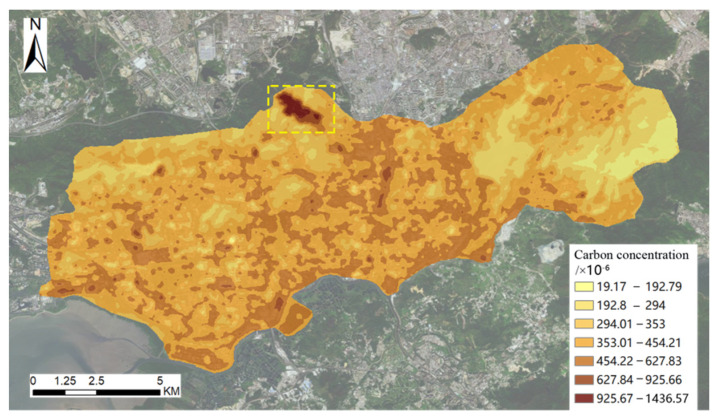
Distribution of CO_2_ concentration in the study area (the yellow dotted line is the abnormal value area).

According to the calculation method in Section 3.3, a linear regression analysis of CO_2_ concentration, Y, and surface temperature, X, is performed. We calculate the regression coefficients of 46.53 and −639, respectively, and the correlation coefficient R is 0.896. Finally, the regression equation obtained is Y = 46.53X − 639. The CO_2_ concentration is calculated by substituting the LST into the formula, as shown in Figure 8. At the same time, we judge the accuracy of the results by random verification. There is only one abnormal value of CO_2_ concentration in the study area (the area within the yellow dotted line in Figure 8). This is the landfill treatment plant. After landfilling, a series of chemical reactions will take place, resulting in a large amount of CO_2_ and heat. Therefore, from a global perspective, the derivation of CO_2_ concentration is relatively referential.

### 4.3. Landscape Index and Its Relationship with CO_2_ Spatial Concentration

**Figure 9:**
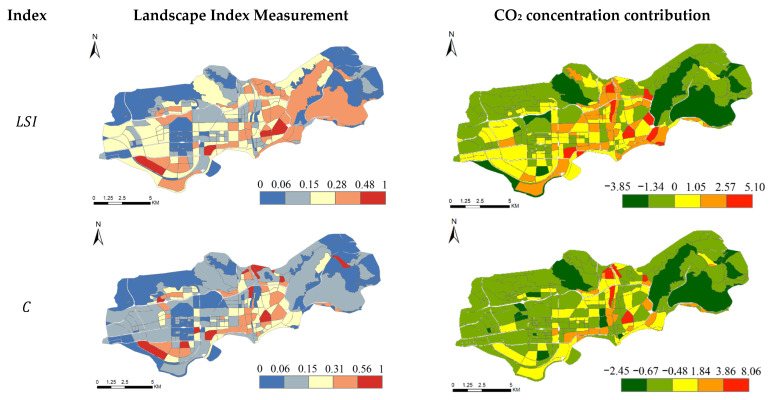

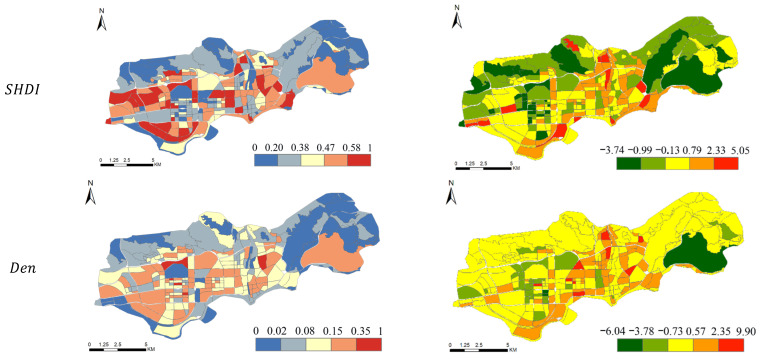
Landscape Index Measurement and CO_2_ concentration contribution.

A structured urban form is the basis of urban development. Urban structure determines urban function, and urban form is the external expression of urban spatial structure. A complete urban plan should include three aspects: urban structure, urban function, and urban form. In order to facilitate the unified calculation of the landscape index, the landscape indices are normalized to analyze the characteristics of urban form and urban function. Figure 9 presents the contribution rate of four landscape indexes and measures and reveals the contribution of different landscape indexes to CO_2_ concentration.

(1) From the perspective of urban form, as seen in the spatial distribution of the LSI index in Figure 9, the urban form is regular in the western region of the study area. In other words, the fractal dimension is low, which verifies the characteristics of heavy “tail” distribution of urban development. The relatively high LSI indices in the eastern part of the study area are a result of the diversity of urban green areas that can be regarded as open spaces. Therefore, it can be seen that the study area has good aesthetic characteristics in urban form design. As the core area of the city, the west of the study area has a wide coverage of public facilities and a low C index. However, in the central part of the study area, the C index is higher because the layout of public facilities is less regular than in other areas.
(2) From the perspective of urban function, most of the SHDI indices in the study area are high, indicating that the overall land use types in the region are diversified. This phenomenon is a result of the western part of the study area being the central business district of Shenzhen, which mainly serves the functions of finance and trade services. It has a large number of amenities such as business offices, hotels, shopping centers, cultural facilities, and high-density public amenities. However, other areas consist mainly of residential, park plazas, and a few commercial functions. Compared to the CBD, they have fewer amenities and therefore have a relatively low Den. It should be noted that there is an orange area in the western part of the study area, which is a relatively independent group with internal facilities that can meet the needs of the residents.

According to the four landscape index measures (Figure 9) and the main functional zoning of the study area (Figure 7), it is believed that an evenly distributed and interconnected landscape structure can effectively perform the ecological and social functions of open space. The analysis shows that in the urban core, a green space structure with a high degree of fragmentation can effectively penetrate the urban space and mitigate the negative ecological effects of the city. Negative ecological effects such as urban heat island and air pollution can be minimized by increasing the patch uniformity and patch density.

The analysis concludes that the stability of the overall ecological quality of the city can be maintained by appropriately increasing the number of small open spaces in the urban center. At the same time, by reducing traffic commuting between individual landscape plots, complete public facilities can be established, thus achieving the goal of reducing greenhouse gas emissions. In conclusion, the overall situation of the study area is a low-carbon sustainable development model with low energy consumption, low pollution, and low emissions. However, regional differences and spatial heterogeneity still exist. Especially in some areas of Luohu District, the efficiency of low-carbon urban development still needs to be improved.

## 5. The Planning Strategy of Low-Carbon Urban

The purpose of shaping “low-carbon urban form” is to reduce urban carbon emissions, which is achieved by shaping and constructing spatial form elements and coordinating and optimizing the interaction mechanisms between them. In order to achieve this goal, we design the urban form to reduce CO_2_ concentration without changing the dominant function. We design the planning scheme from the three aspects of optimal form, optimal function, and comprehensive development, and estimate the possible characteristics of CO_2_ concentration changes caused by each simulation scheme.

**Figure 10:**
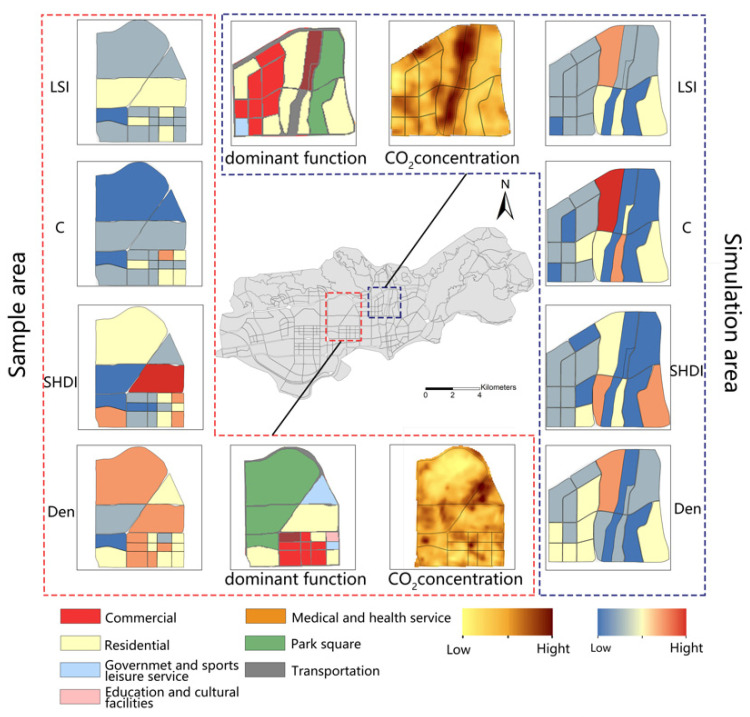
Current situation of landscape indices in sample area and simulation area.

Based on the CO_2_ spatial concentrations plotted in Section 4, the red-boxed area in Figure 10, located near the central business district of Futian District, is selected as the sample area. This region is characterized by a complete urban function and low CO_2_ concentration. Similarly, the other part of the continuous landscape parcels with high total CO_2_ in Luohu District is selected as the simulation area (blue frame area in Figure 10). This region still has high CO_2_ emissions in the case of more green space. Without changing the dominant function, the detailed schemes are described as follows.

**Figure 11:**
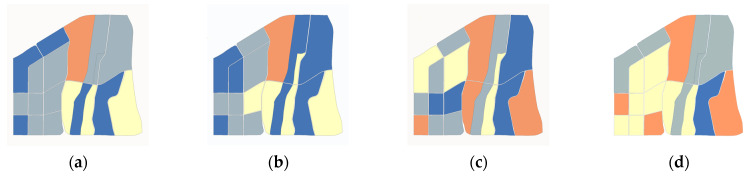
Optimized landscape index. (**a**) LSI. (**b**) C. (**c**) SHDI. (**d**) Den.

The four low-carbon landscape indices are depicted in Figure 11 as a reference for the subsequent calculation of the optimal planning scheme. Our scheme is divided into three modes: optimal morphology, optimal function, and comprehensive development.

**Figure 12:**
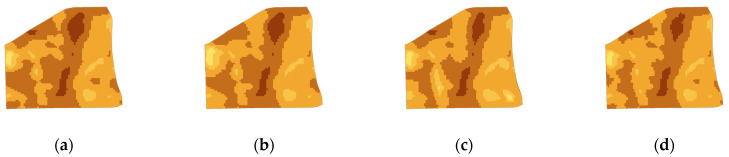
Simulation diagram of CO_2_ concentration distribution under different schemes. (**a**) current situation. (**b**) experiment A. (**c**) experiment B. (**d**) experiment C.

(1) Optimal morphology: The LSI and C are effective indicators to reflect the urban morphology. The lower these values are, the more regular and stable the urban form is. Therefore, according to the requirements of reducing LSI and C, the industrial layout modes of different business types have been adjusted. Through homogenization and standardization of facility layout, landscape fragmentation in the simulation area is reduced. The lower the mixing degree of regional functions, the more obvious is the improvement of landscape compactness after the regulation. Therefore, this scheme can locally reduce the CO_2_ concentration and is more suitable for areas with a low functional mixing degree. By reducing LSI by 6.1% and C by 9.4%, the carbon reduction ratio of this scheme is 14.31% (Figure 12b).
(2) Optimal function: SHDI and Den reflect function diversification and spatial agglomeration characteristics, respectively. Therefore, by increasing the low-energy consumption business types and adding public facilities appropriately in the simulation area, SHDI and Den can be increased. Notably, this scheme is suitable for old urban areas that cannot be demolished and built, and which can reduce CO_2_ concentration globally. In this scheme, LSI is reduced by 16.3% and C is reduced by 12.1%, resulting in a 15.15% carbon reduction ratio (Figure 12c).
(3) Comprehensive development: To achieve the goal of comprehensive development, the morphology and function of the simulation area are regulated and adjusted simultaneously. In this scheme, this is carried out by rebuilding existing buildings and improving infrastructure to adjust LSI, C, SHDI, and den at the same time. It is suitable for new urban districts with unused land or suburban areas, and its CO_2_ concentration reduction efficiency is the highest, reaching 27.72% (Figure 12d).

## 6. Conclusions and Discussions

### 6.1. Conclusions

The Low-carbon development of cities has played an important role in global carbon emission reduction, and urban morphology can affect carbon emissions through energy use, transportation, and other factors. Thus, which types of urban form and function combinations can reduce carbon emissions is a question worth discussing. To explore this issue, the following research was carried out:

(1) Based on the unique law of heavy “tail” distribution in urban development, the head/tail breaks method under the third fractal theory came into being. We use this method to divide the evaluation parcels of Futian District and Luohu District, which ensures the integrity of the landscape morphology.
(2) To identify urban functions, semantic association and the LSTM model are integrated. Then, landscape ecological theory is introduced to calculate the landscape index based on POI and OSM data, and this is used to measure the urban morphology. It is found that urban form design is regular and urban land use types are diverse in the study area, and the spatial distribution pattern of urban public service facilities is reasonable, which makes the study area a livable city.
(3) The LST is inversed by Landsat-8 remote sensing data, and the relationship equation between it and the actual CO_2_ concentration is established to estimate the CO_2_ concentration in the study area. Then, we quantitatively analyze the contribution of different landscape indices to carbon emissions. The analysis shows that a green space structure with uniform distribution and close connection can give full play to the ecological and social functions of green space. It is considered that the open space structure, with strong accessibility and high density, penetrates into the urban core area to alleviate the negative ecological effects of the city. In addition, at the outer edge of the city, large-scale green space can strengthen its ecological function.

In summary, regular form and perfect infrastructure are conducive to reducing carbon emissions. Therefore, the relationship between urban spatial form and carbon emissions is an important basis for spatial planning, government decision-making, and sustainable development.

### 6.2. Discussions

To build a low-carbon city, spatial planning needs to add new carbon neutralization dimensions and weights for considering and evaluating spatial planning schemes. Traditional data sources have the disadvantages of poor real-time effect, they are difficult to obtain, and have low accuracy, and they cannot meet this goal. Therefore, the multi-source spatio-temporal data represented by POI and OSM data is applied to describe the urban landscape morphology. However, this study inevitably has many limitations, and further investigation is needed:

(1) The CO_2_ concentration cannot completely fit the carbon flux in the region. Hence, it is necessary to explore the relationship between CO_2_ concentration and carbon flux.
(2) Owing to the different plant types and climates in different regions, the experimental results may be different. Therefore, regional factors need to be considered in future research.
(3) Other factors, such as population distribution and energy consumption demand, may have a significant impact on carbon dioxide emissions. Therefore, social impact factors need to be considered in future research.

## Figures and Tables

**Table 1:** List of data sources.

Type of Data	Data Sources	Data Usage	Time of Data
POIs data	https://lbs.amap.com/ (accessed on 1 January 2022)	Dominant function identification and landscape index calculation	2022
OpenStreetMap	https://www.openhistoricalmap.org/ (accessed on 1 January 2022)	Division of evaluation parcels	2022
Landsat-8 image	https://www.nasa.gov/ (accessed on 20 February 2021)	CO_2_ concentration calculation	2021

**Table 2:** POIs function classification table.

Function Type	Corresponding POI Classification
Commercial	Restaurant, cafe, bar, tea house, hotel, cinema, shopping mall, shopping center, wholesale market, monopoly store, supermarket, convenience store, home building materials store, digital appliance, market, shop, beauty salon, barbershop, manicure, resort, KTV, theater, dance hall, internet bar, playground, bathing and massage, leisure plaza, company, bank, insurance, business hall, training institution, bookstore, gas station, driving school (registration offices), pet hospital, printing shop
Residential	Residence, apartment, dormitory, villa
Government and sports leisure service	Government, fire department, police station, court, industry and commerce bureau, tax bureau, finance bureau, customs, embassy, association, foundation, welfare institution, stadium, swimming pool, basketball court, badminton court
Education and cultural facilities	Kindergarten, nursery school, primary school, middle school, high school, university, adult education, vocational school, special education school, private school, scientific research institution, museum, library, conference center
Medical and health service	Hospital, children’s hospital, clinic, emergency center, psychiatric hospital, infectious disease hospital, tuberculosis, eye hospital
Park Square	Park, green space, botanical garden, water
Transportation	Bus station, high-speed train station, railway station, subway station, driving school (training field)
Industrial	Factory, processing plant, logistics, warehousing, water purification plants
Other	Graveyard, public toilet, parking lot, water supply, electricity, garbage disposal site, temple

**Table 3:** Landscape index calculation.

Landscape Index	Calculation Formula	Description
Landscape Shape Index	$LSI=\frac{Sum\_L_{i}}{2\sqrt{\pi A_{i}}}$	It reflects the fractal dimension and morphological characteristics of urban landscape elements.
Fragmentation Index	$C=\frac{N_{i}}{A_{i}}\times \frac{k_{i}}{K}$	It reflects the degree of fragmentation of landscape elements
Shannon’s Diversity Index	$SHDI=-\sum_{j=1}^{k}P_{ij}\ln\left(P_{ij}\right)$	It reflects the diversity of urban public facilities.
Density of Public Facilities	$Den=\frac{F_{i}}{A_{bi}}$	It reflects the density of urban public facilities.

LSI is the Landscape Shape Index of parcel i; $Sum\_L_{i}$ is the sum of the boundary lengths of landscape patches in parcel i; $A_{i}$ is the area of AOI in parcel i; C is the Fragmentation Index of parcel i; $N_{i}$ is the number of patches in parcel i; $k_{i}$ is the number of POI types in parcel i; K is the total number of POI categories; SHDI is the Shannon’s Diversity Index of parcel i; $P_{ij}$ is the proportion of the number of type j POIs in all POIs in parcel i; k is the number of POI types in parcel i; Den is the Density of Public Facilities in parcel i; $F_{i}$ is the number of public facility POIs in parcel i; $A_{bi}$ is the area of the building in parcel i; i is the ID of the evaluation parcel.

**Table 4:** Statistics of POI and landscape dominant function parcel.

ID	Type	POI Number	Weight	Number of Functional Parcels	Functional Parcel Area/km^2^
1	Commercial	194,970	1.834312	65	17.4
2	Residential	25,569	1.640193	100	55.27
3	Government and sports leisure service	5811	2.648612	10	3.13
4	Education and cultural facilities	7506	2.438533	21	6.81
5	Medical and health service	2710	2.461524	2	0.81
6	Park Square	971	2.158362	67	54.43
7	Transportation	5841	2.306906	8	16.78
8	Industrial	4234	1.539243	10	3.91
9	Other	1254	2.153471	4	2.47
